# Treatise on Hare Lip

**Published:** 1845-12-01

**Authors:** 


*Treatise on Hare-lip, by Dr. S. P. Hullihen, of Wheeling, Va.*—This is a pamphlet of 12 pages, for a copy of which we are indebted to the author. His object is to recommend the application of adhesive plasters sufficiently broad to cover the cheeks, connected with straps passing over the lip, to remedy the cleft in the alveolar and palatine arches when this occurs as a complication of hare-lip. He states that if this measure be adopted soon after birth, the bones are brought readily into apposition, and the deformity removed. He adopts the same means to secure union of the hare-lip after operating in the usual way.

